# Monitoring cognition in multiple sclerosis via adaptive smartphone games—first insights from a validation study

**DOI:** 10.3389/fdgth.2026.1627226

**Published:** 2026-02-10

**Authors:** Silvan Pless, Tim Woelfle, Johannes Lorscheider, Andrea Wiencierz, Óscar Reyes, Carlos Luque, Pasquale Calabrese, Cristina Granziera, Ludwig Kappos

**Affiliations:** 1 Research Center for Clinical Neuroimmunology and Neuroscience Basel (RC2NB), University Hospital Basel, University of Basel, Basel, Switzerland; 2 Neuropsychology and Behavioral Neurology Unit, Department of Psychology and Interdisciplinary Platform Psychiatry and Psychology, Division of Molecular and Cognitive Neuroscience, University of Basel, Basel, Switzerland; 3 Multiple Sclerosis Center, University Hospital Basel, Basel, Switzerland; 4 Department of Neurology, University Hospital Basel, Basel, Switzerland; 5 Translational Imaging in Neurology (ThINK) Basel, Department of Biomedical Engineering, Faculty of Medicine, University Hospital Basel, University of Basel, Basel, Switzerland; 6 Reha Rheinfelden, Rheinfelden, Switzerland; 7 Department of Clinical Research, University Hospital Basel, University of Basel, Basel, Switzerland; 8 Indivi AG, Basel, Switzerland

**Keywords:** cognitive assessment, cognitive impairment, digital biomarkers, gamification, monitoring tool, multiple sclerosis, smartphone-games

## Abstract

Cognitive impairment in Multiple Sclerosis significantly undermines quality of life and working capacity yet despite its high prevalence (30%–75%) it is often neglected. Gamified and adaptive smartphone tests have great potential to assess cognition reliably and conveniently. To improve monitoring of cognition in people with MS (pwMS) we developed CoGames, a set of 6 adaptive and gamified smartphone tests. Here we present first insights into their reliability, correlations to domain-corresponding reference tests, and adherence. The games were played repeatedly over 6 weeks. We calculated correlation estimates between game-derived measures of the first two runs for the test-retest reliability analysis, correlated (partial Spearman correlation) game measures with the results of established reference tests and assessed adherence as the proportion of played- to scheduled games. We included data collected between March 2022 and October 2023 of the first 100 pwMS, mean age: 46.8 (±12.3), median EDSS: 2.5 (0–7.0), who completed the initial intensive phase of the dreaMS validation study 1 (NCT05009160). Test-retest correlation estimates ranged from r = 0.41 to 0.91. Correlation estimates to reference tests ranged from |ρ| = 0.23 to 0.66. Average adherence was 89.3% (86%–92%). Our results suggest high test-retest reliability of the CoGames and clear correlations with their reference tests. Adherence was higher than for other tests included in the dreaMS app underlining the value of gamification. Albeit these results must be confirmed in a larger population over a longer time, they support the potential of CoGames as a valid, reliable and enjoyable monitoring-tool for cognition in pwMS.

## 1 Introduction

Multiple Sclerosis (MS) is an inflammatory and neurodegenerative auto-immune disease of the central nervous system (1, 2). Worldwide an estimated 2.8 million people are affected by MS and its heterogeneous manifestation of symptoms including fatigue, impairments in motor- and cognitive functions, and somatosensory dysfunctions, among others (3, 4). Cognitive impairment is common in MS, with a prevalence of 30%–45% in relapsing-remitting MS and 50%–75% in secondary progressive MS (5). Despite the high prevalence and the negative impact on quality of life and working capacity, current neuropsychological assessment is still expensive, requires trained neuropsychologists, and is often not well-accepted by people with MS (pwMS) (6).

To address these shortcomings, a more convenient and personalized monitoring method for cognition is needed. Smartphone apps have great potential in assessment and training of cognitive functions thanks to their user-friendliness, accessibility, accuracy, and objectivity (7–20) Consequently, interest in gamified cognitive assessments and training in the medical field has increased in recent years (21–24) Gamification elements can be rewarding and motivational, making tasks more enjoyable (25–27) Furthermore, a dynamic difficulty adjustment system (DDA) can circumvent boredom (task too easy) and frustration (task too difficult) and lower the risk of floor or ceiling effects (28, 29). Additionally, an adaptive difficulty promotes the flow state, a state of high concentration (28–32). Such attributes are promising to establish high adherence especially in long-term monitoring of cognitive function of people with chronic diseases. Beyond the flow-state, gamification elements might also increase neural engagement. Neuroimaging studies using functional magnetic resonance imaging and functional near-infrared spectroscopy have shown that gamified cognitive tasks modulate attentional and prefrontal control networks and enhance activation in reward- and emotion- related brain regions (33, 34). Furthermore, an electroencephalography study reported increased right-parietal theta power in participants completing a gamified n-back task compared with participants completing a non-gamified version, a pattern commonly associated with greater cognitive effort and sustained attention (35). These findings suggest that gamification may also enhance neural mechanisms underlying effort and concentration—two key drivers of performance in cognitive assessment and training. Such effects may be particularly relevant in MS, where cognitive fatigue is highly prevalent and represents a major contributor to reduced cognitive performance.

In cooperation with the medical software company Indivi AG (https://www.indivi.health), we have developed the dreaMS app, an application comprising multiple tests assessing key functional domains for MS including movement-, balance-, vision-, and cognitive tests (16). To validate this app as a smartphone-based monitoring tool for disease activity-and progression in pwMS, we are conducting the dreaMS Validation study 1 (NCT05009160), a multi-center study nested in the Swiss MS Cohort Study (SMSC). The cognitive tests (CoGames) include elements of gamification and adapt their level of difficulty to the users performance. In a previous study with a cohort of healthy volunteers we were able to show high acceptance and reliability for this set of 6 smartphone-based and gamified cognitive tests (36).

In this data look analysis, we used data collected from the first 100 patients during the first 6 weeks of the study, aiming at exploring the reliability of the CoGames as well as correlation estimates to domain-corresponding established neuropsychological tests (NPT), and adherence. This is an exploratory analysis refraining from addressing statistical hypotheses and from reporting measures of significance.

## 2 Methods

### 2.1 Standard protocol approvals, registrations, and patient consents

This paper reports results from a data look of the on-going study: “DreaMS—Development of Digital Biomarkers in Multiple Sclerosis Validation Study 1”. The dreaMS VS1 is being conducted according to the standards of the World Medical Association Declaration of Helsinki and was approved by the local ethics committee: Ethikkommission Nordwest- und Zentralschweiz (EKNZ), Basel, Switzerland (BASEC ID 2021 D0040). It is registered at clinicalTrials.gov: NCT05009160, and at the Swiss National Clinical Trials Portal (SNCTP000004678). All participants were informed about all relevant aspects and risks of the study and were required to sign an informed consent form (ICF).

### 2.2 Study population and procedures

For this study we used data from a planned data-look that includes the first 100 pwMS who completed the first 6 weeks of the dreaMS VS1. We included our first patient on March 30^th^ 2022. Inclusion criteria were: Age ≥18, a diagnosis of MS including all clinical forms (CIS, RRMS, SPMS, PPMS) according to the revised McDonald criteria 2017, possession of an dreaMS app-compatible smartphone (iOS/Android), corrected close visual acuity of ≥0.5, hand motor skills sufficient for using a smartphone, ability to follow the study procedures, and informed consent as documented by signature. Exclusion criteria comprised other clinically significant concomitant disease states (e.g., renal failure, severe hepatic dysfunction, severe/unstable cardiovascular disease, progressive cancer, etc.), and known or suspected non-compliance.

At the baseline-visit all participants were instructed on how to download and use the dreaMS app on their personal smartphone. All study participants completed all dreaMS tests once in-clinic under supervision of a study nurse. After the baseline-visit, participants completed every dreaMS tests once per week for the first 6 weeks (intensive phase), then once per month for the rest of the study, with recurring 4-week intensive phases before and after in-clinic follow-up visits at 12 and 24 months. Participants were provided a list of tests to complete in the week to come and were allowed to choose the day, time, and location to complete the tests within this week.

### 2.3 Instruments and measures

**Figure 1:**
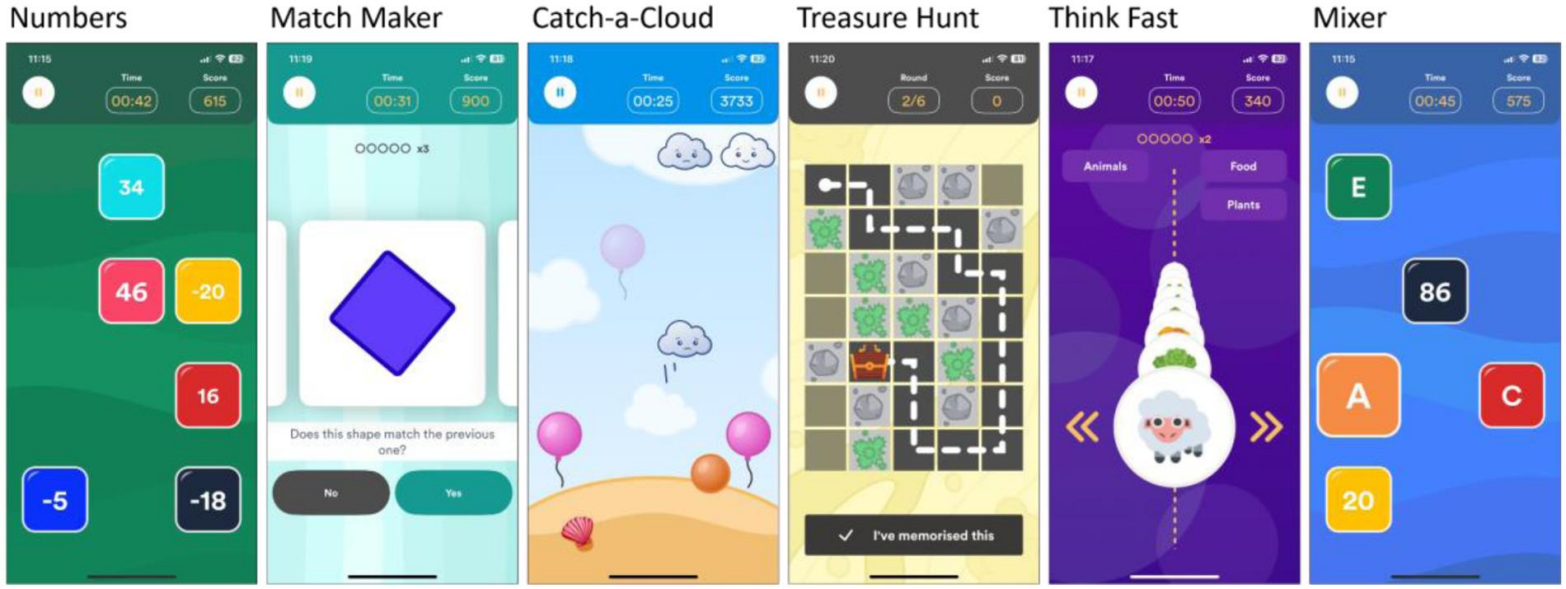
Screenshots of the CoGames in *adaptive mode.*

The CoGames are a set of 6 smartphone-games based on established NPTs with the aim of monitoring cognitive function in a convenient and enjoyable manner. Figure 1 shows a screenshot of the CoGames. Every game assesses a cognitive domain frequently affected in MS: psychomotor speed, working memory, visual short-term memory, mental flexibility, and information processing speed (IPS). For IPS, the most prominently affected cognitive domain in MS, we included 2 games with slightly different assessment approaches. An overview of all games, their corresponding cognitive domains, predefined measures, and short descriptions is shown in Table 1. We developed two “Modes” of each game: *Fixed Mode* and *Adaptive Mode*. The *Fixed Mode* remains unchanged over time and—to prevent ceiling effects—includes multiple difficulty levels within one run. Participants are asked to play the *Fixed Mode* only at baseline, M6, M12, M18, and M24 to minimize learning effects. In most games the main measure in the *Fixed Mode* is “completion time”. Rather than having fixed items for every run like the *Fixed Mode*, the *Adaptive Mode* has established difficulty variables which determine the then randomized items. For example, in the game *Numbers*, where the user must tap on numbers in ascending order, the *Fixed Mode* uses the same numbers in every run, while the *Adaptive Mode* randomizes the numbers stratified by difficulty ranges (e.g., Level 1: from −25 to 25, Level 2: from −45 to 60, etc.). This randomized approach allows the games to be played at a higher frequency without the risk of learning effects through recall of specific items. Contrary to the *Fixed Mode*, the *Adaptive Modes* difficulty-level adapts only if a certain threshold of game score is reached in two consecutive runs. This dynamic difficulty adjustment system allows an on-going adaptation of difficulty to the patient's performance. This helps to avoid boredom and ceiling effects or frustration and floor effects by tasks being too easy or too difficult, respectively. The number of levels ranges from 3 to 6, depending on the game. To keep the monitoring as convenient as possible, the *Adaptive Mode* is limited to 1 minute, except for the short-term memory game, where a time-factor might have biased the assessment of memory. The primary measures are therefore “number of correct responses” or “percentage of correct responses”, depending on the game. The psychomotor game *Catch a Cloud* has a slightly different *Fixed* vs. *Adaptive Mode* design compared to the other games. Here every run includes both modes. The first 20 seconds are played in *Fixed Mode* immediately followed by 30 seconds of *Adaptive Mode*. An overview of the characteristics of the *Fixed Mode* and *Adaptive Mode* can be found in Table 2.

**Table 1:** Cognitive domains, games, measures, description of the games, reference tests.

Cognitive domain	Game (No. of levels in the *fixed mode*)	Primary measures (*adaptive mode*/*fixed mode)*	Description	Reference tests (main measure)
Working memory	Match maker (3 levels)	Number of correct responses in 60 sec./completion time	The system shows an either colorful or grey shape on the screen. With every answer, a new shape appears and the previous is hidden. The task is to continuously decide (“Yes”/“No”) whether the presented shape matches the one shown “n” taps before. “N” increases with higher difficulty.	Backwards digit span (44) (number of correct responses; max: 12 points)
Information processing speed	Think fast (4 levels)	Number of correct responses in 60 sec./completion time	The system shows an image which must be sorted into the correct category as fast as possible. The number of categories and their similarity change with difficulty.	Trail making test A (45) (completion time); oral Symbol Digit Modalities Test (46) (number of correct responses in 90 seconds)
	Numbers (3 levels)	Number of correct responses in 60 sec./completion time	The system displays a series of numbers that are randomly placed across the screen. The participant's task is to tap the numbers in ascending order, as fast as possible.	Trail making test A (45) (completion time); oral symbol digit modalities test (46) (number of correct responses in 90 seconds)
Short-term memory	Treasure hunt (4 levels)	Mean percentage of correct grid-square connections over 6 rounds	The system shows a grid with a path to an “X” (treasure). The participant must memorize the exact path to the treasure. After an intermission time the participant must reconstruct the memorized path on a blank grid. Grid size, path length and intermission time length are increased with higher difficulty.	Rey Osterrieth Complex Figure Test—immediate & delayed recall (47) (score of correctly remembered elements of a complex geometrical figure after 3- and 30 minutes; max: 36 points per subtest)
Mental flexibility	Mixer (3 levels)	Number of correct responses in 60 sec./completion time	The system displays a series of letters and numbers that are randomly placed across the screen. The participant must tap the numbers and letters in ascending-/ alphabetical order, as fast as possible, always alternating between the two.	Trail making test B (45) (completion time)
Psychomotor speed	Catch a cloud (6 levels)	Number of correct responses in 50 sec.(*Fixed Mode*: 20 sec.,*Adaptive Mode*: 30 sec.)	Clouds on screen must be “tapped” as fast as possible. Once tapped, the cloud reappears at another location on the screen. The first 20 seconds (*Fixed Mode*) only shows 1 cloud on screen. After the *Fixed Mode*, the *Adaptive Mode* follows for 30 seconds. Higher levels of the *Adaptive Mode* include more gamification elements such as raining clouds, distractions (Balloons) which must be avoided, and multiple clouds at once.	Trail making test A (45) (completion time); oral symbol digit modalities test (46) (number of correct responses in 90 seconds)

**Table 2:** Description of fixed mode and adaptive mod*e.*

	*Fixed mode*	*Adaptive mode*
Schedule	Is played at the baseline visit in the clinic under supervision and then every 6 months remotely.	Weekly for the intensive phase (first 6 weeks). Then monthly where games are split into weekly chunks. The day and time are free to choose by the participant. Around the yearly follow-up visits a 4-week intensive phase is repeated, where the games are played weekly.
Data included in this analysis	Only *Fixed mode* data of the baseline visit was used in this analysis.	The average scores of the *Adaptive modes* run 1 and 2 were used in the analyses. This ensured that all patients played the same difficulty level.
Measure	Completion time, percent of correct connections (*Treasure Hunt*), number of correct responses in 20 seconds (*Catch a Cloud*).	Number of successful responses in 60 seconds, percent of correct connections (*Treasure Hunt*), number of successful responses in 30 seconds *(Catch a Cloud*).
Gameplay	A fixed number of rounds with the same items/paths. All items/paths are exactly the same (fixed) at every iteration.	Fixed time (generally 60 seconds and 30 seconds in *Catch a Cloud*) or fixed number of rounds (*Treasure Hunt*). The items/paths of all rounds are randomized according to the difficulty level.
Difficulty	Increases gradually, includes all difficulty levels.	The same during an iteration. Adaptive depending on performance: if in 2 consecutive iterations a threshold is reached, the patient levels up-/ down.

### 2.4 Objectives and outcomes

Our objective was to investigate the test-retest reliability, concurrent validity, and adherence of the CoGames as an assessment tool for cognitive function in MS. Hence our outcomes were (a) Pearson or Spearman correlation coefficients, depending on the game measures distribution, with 95% confidence intervals for the test-retest reliability analysis; (b) cross sectional point estimates as partial spearman correlation coefficients controlled for age, sex, and operating system with 95% confidence intervals between the primary CoGames measures (*Fixed Mode* & *Adaptive Mode* separately) and their predefined reference tests; and (c) the level of adherence in percentage (total completed/total scheduled) compared to the less gamified tests included in the dreaMS app.

### 2.5 Data collection and statistical analyses

Data collection, data cleaning and feature extraction processes were performed using Python 3.9 coding language. All statistical analyses were performed using R version 4.4.0 (24.04.2024). To estimate reliability, we used Pearson and, for skewed distributions, Spearman correlations, with their 95% confidence intervals between the first two runs of the *Adaptive Mode*. Since changing levels requires reaching a threshold in two consecutive runs, focusing on only the first two runs ensured that all participants were still on the same difficulty level, thus creating a clean test-retest situation. The statistical analysis methods for correlations between CoGames-measures and reference tests (partial Spearman correlations) was the same for the *Fixed Mode* and the *Adaptive Mode*. However, for the *Adaptive Mode* we used the average score of the first 2 runs for the analyses. This limitation to only 2 runs was necessary since including more runs would have meant that individuals might have been in different difficulty levels, making it difficult to compare the raw scores. The partial Spearman correlations were controlled for age, sex, and operating system by first rank-transforming the data and then using a regression residualization, where the residuals of all variables (dependent and independent) were correlated (Pearson correlation) using the “pcor.test” function of the R package “ppcor”. Since the CoGames were intentionally designed to be accessible for individuals with fine-motor impairments, requiring only gross hand movements rather than precise finger control, and our reference tests included oral and written tests, we did not additionally control for dexterity or other motor functions. Mean adherences for all CoGames and the other dreaMS tests is reported as percentages (total completed/total scheduled).

## 3 Results

Seventy-two (72%) participants were women, the mean age 46.8 (±12.3) and median Expanded Disability Status Scale (EDSS) 2.5 (range 0–7.0) (37). Based on the baseline screening with the Multiple Sclerosis Inventory for Cognition (MUSIC) 87% of the patients had no signs of cognitive impairment, 9% showed mild-, 3% moderate-, and 1% severe cognitive dysfunctions (38). The operating systems used in this analysis were iOS: 60 (60%), Android: 40 (40%) and the main brands were Apple, Samsung, Huawei, and to a lesser degree Xiaomi, Sony, Pixel, Nokia, Motorola, Fairphone, Cubot. An overview of patient demographics is shown in Table 3.

**Table 3:** Participants demographics.

PwMS	*N* = 100
Mean age in years (SD), range	46.8 (±12.3)23–76
Sex (%)	Female 72 (72%)
Operating system (%)	iOS = 60 (60%), Android = 40 (40%)
Smartphone brands	Apple, Samsung, Huawei, Xiaomi, Sony, Pixel, Nokia, Motorola, Fairphone, Cubot
MS-Course, n (%)	CIS: 9 (9%)
	RRMS: 86 (86%)
	PPMS: 3 (3%)
	SPMS: 2 (2%)
Disease duration in years, median (range)	12.2 (0.2–37.8)
Median MUSIC score (range) MUSIC score distribution *n* (%)	26 (10–32)
	No cognitive impairment: 87 (87%)
	Mild cognitive impairment: 9 (9%)
	Moderate cognitive impairment: 3 (3%)
	Severe cognitive impairment: 1 (1%)
Median EDSS (range)	2.5 (0–7.0)

### 3.1 Test-retest reliability

We used Pearson correlations between the first 2 *Adaptive Mode* runs for most of the test-retest analyses. For *Treasure Hunt* and *Catch a Cloud*, Spearman correlation was used because of their skewed distributions. Exploratorily, for *Treasure Hunt*, we investigated secondary performance measures. An overview of all reliability measures is shown in Table 4.

**Table 4:** Pearson test-retest correlation coefficient estimates between the first 2 runs of all CoGames in the adaptive mode and their 95% confidence interval (spearman correlation for skewed distributions).

Game	Measure	Pearson correlation [95% CI]	*n*
Match maker	Successful responses in 60 seconds	0.91 [0.87, 0.94]	98
Think fast	Successful responses in 60 seconds	0.9 [0.86, 0.93]	98
Numbers	Successful responses in 60 seconds	0.79 [0.7, 0.86]	98
Treasure hunt	Mean % of correct connections over 6 rounds Time used for memorization Total completion time (memorization & recall)	Spearman's rho: 0.41 [0.23, 0.58] Spearman's rho: 0.75 [0.64, 0.83] Spearman's rho: 0.71 [0.59, 0.81]	97
Mixer	Successful responses in 60 seconds	0.87 [0.82, 0.91]	99
Catch a cloud	Successful responses in 30 seconds	Spearman's rho: 0.86 [0.77, 0.92]	99

### 3.2 Correlation between CoGames measures and reference tests

#### 3.2.1 Fixed mode

**Figure 2:**
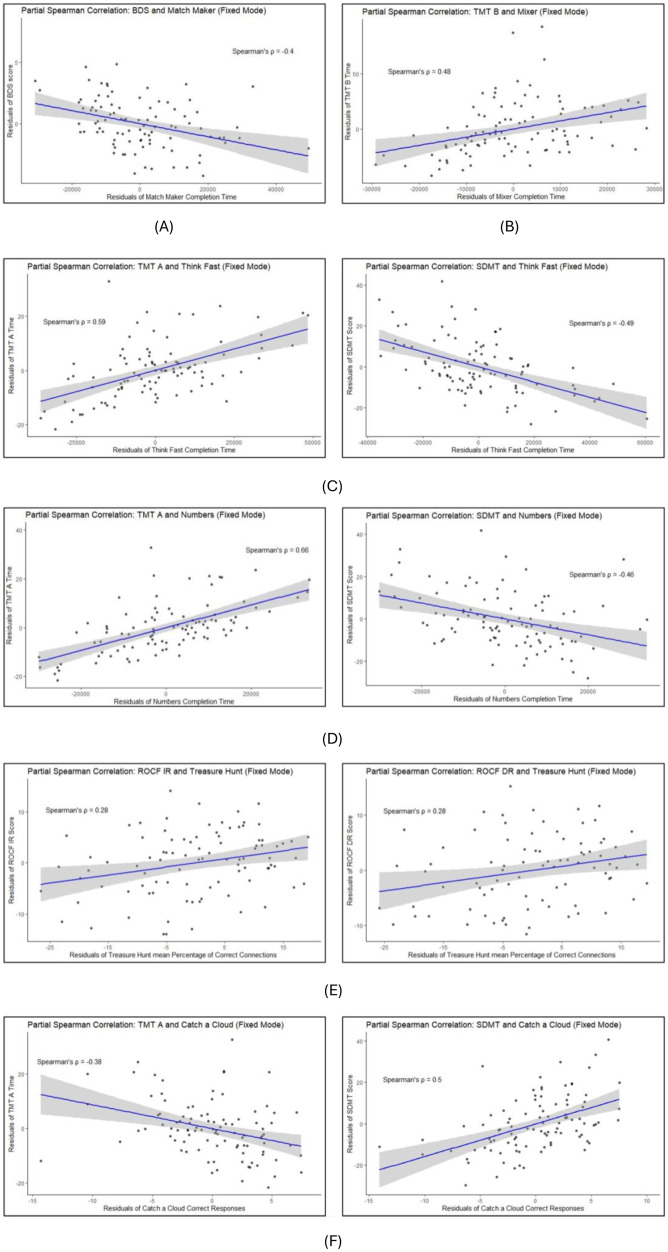
Correlations between CoGames and reference tests in *fixed mode.* **(A)** Match maker vs. backwards digit span. **(B)** Mixer vs. trail making test B. **(C)** Think fast vs. trail making test A and symbol digit modalities test. **(D)** Numbers vs. trail making test A and symbol digit modalities test. **(E)** Treasure hunt vs. Rey-Osterrieth complex figure test immediate- and delayed recall. **(F)** Catch a cloud vs. trail making test A and symbol digit modalities test.

Age-, sex-, and operating system-corrected partial spearman correlation estimates between the primary CoGames measures in *Fixed Mode* and their domain-corresponding reference test ranged from |*ρ*| = 0.28 to 0.66 (see Table 5 and Figure 2).

**Table 5:** Partial spearman correlation coefficients estimates and 95% confidence intervals between primary CoGames measures in the fixed mode and reference tests.

Game (measure)	Domain	Reference test	Partial Spearman correlation [95% CI]	*n* PwMS
Match maker (completion time)	Working memory	BDS Score	−0.4 [−0.56, −0.21]	93
Think fast (completion time)	Information processing speed	TMT A SDMT	0.59 [0.43, 0.71] −0.49 [−0.63, −0.32]	95 95
Numbers (completion time)	Information processing speed	TMT A SDMT	0.66 [0.53, 0.76] −0.46 [−0.61, −0.28]	95 95
Treasure Hunt (mean % of correct connections over 6 rounds with increasing difficulty)	Short-term memory	ROCF-IR ROCF-DR	0.28 [0.07, 0.46] 0.28 [0.07, 0.47]	92 84
Mixer (completion time)	Mental flexibility (set-shifting)	TMT B	0.48 [0.3, 0.62]	96
Catch a Cloud (number of correct responses in 20 seconds)	Psychomotor speed	TMT A SDMT	−0.38 [−0.55, −0.2] 0.5 [0.33, 0.64]	97 97

BDS, backwards digit span; TMT A & B, trail making test A&B; SDMT, symbol digit modalities test; ROCF-IR, Rey-Osterrieth complex figure—immediate recall; ROCF-DR, Rey-Osterrieth complex figure—delayed recall.

Exploratorily, we conducted partial spearman cross-correlations between the primary CoGames measures and all reference tests measures including the 95% confidence intervals. Heatmaps containing the cross-correlation matrices are shown in the supplementary material (Supplementary Figure S2).

#### 3.2.2 Adaptive mode

**Figure 3:**
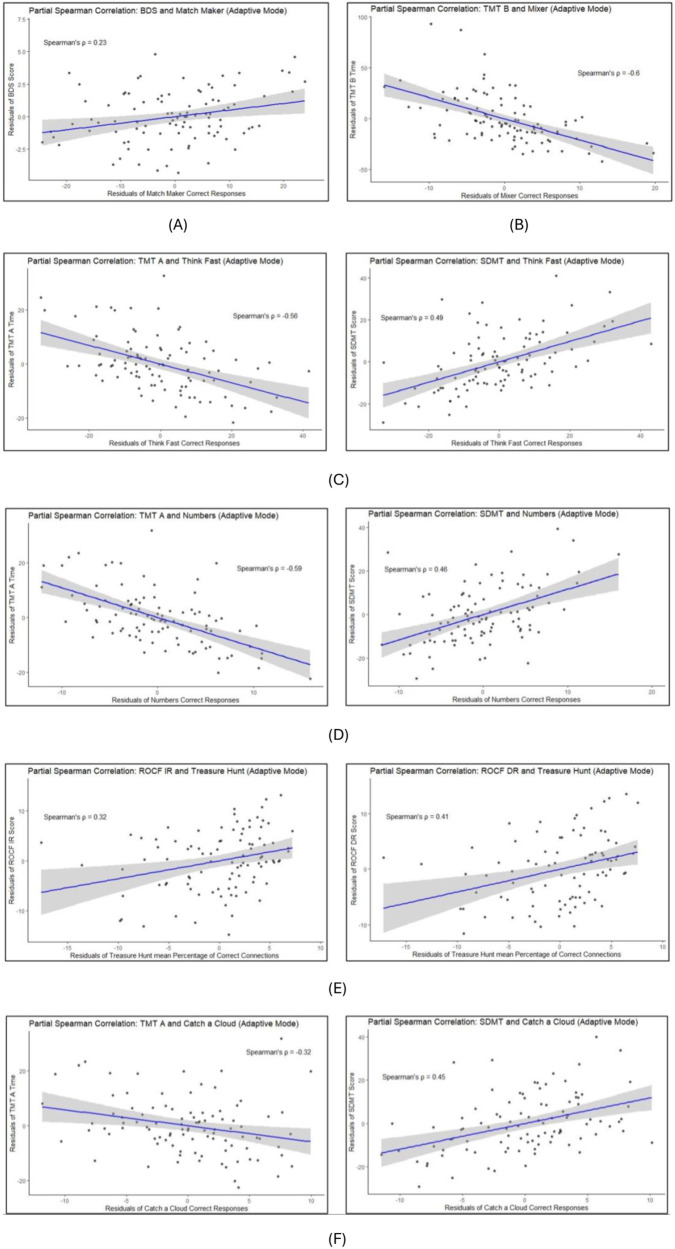
Correlations between CoGames and reference tests in *adaptive mode.* **(A)** Match maker vs. backwards digit span. **(B)** Mixer vs. trail making test B. **(C)** Think fast vs. trail making test A and symbol digit modalities test. **(D)** Numbers vs. trail making test A and symbol digit modalities test. **(E)** Treasure hunt vs. Rey-Osterrieth complex figure test immediate- and delayed recall. **(F)** Catch a cloud vs. trail making test A and symbol digit modalities test.

In the *Adaptive Mode*, the age-, sex-, and operating system-corrected spearman correlation estimates ranged from |ρ| = 0.23 to 0.60 (see Table 6 and Figure 3).

**Table 6:** Partial spearman correlation coefficient estimates and 95% confidence intervals between primary CoGames measures in the adaptive mode and reference tests.

Game (measure)^a^	Domain	Reference test	Spearman correlation [95% CI]	*n* PwMS
Match maker (successful responses)	Working memory	BDS score	0.23 [0.02, 0.41]	97
Think fast (successful responses)	Information processing speed	TMT A SDMT	−0.56 [−0.69, −0.41] 0.49 [0.32, 0.63]	98 98
Numbers (successful responses)	Information processing speed	TMT A SDMT	−0.59 [−0.71, −0.44] 0.46 [0.29, 0.61]	97 97
Treasure hunt (mean % of correct connections over 6 adaptive rounds)	Short-term memory	ROCF IR ROCF DR	0.32 [0.13, 0.5] 0.41 [0.21, 0.57]	95 87
Mixer (successful responses)	Mental flexibility (set-shifting)	TMT B	−0.6 [−0.72, −0.45]	98
Catch a cloud (successful responses in 30 seconds)	Psychomotor speed	TMT A SDMT	−0.32 [−0.5, −0.13] 0.45 [0.26, 0.6]	97 97

a Average of the first 2 runs; BDS, backwards digit span; TMT A&B, trail making test A&B; SDMT, symbol digit modalities test; ROCF-IR, Rey-Osterrieth complex figure—immediate recall; ROCF-DR, Rey-Osterrieth complex figure—delayed recall.

Equivalent to the *Fixed Mode* analyses we exploratorily investigated partial spearman cross-correlations including all reference tests and all game measures. The complete correlation matrices including 95% CI between the games in *Adaptive Mode* and all reference tests are shown as heatmaps in the Supplementary Material (Supplementary Figure S3).

### 3.3 Associations between the fixed mode and the adaptive mode

Exploratorily, we investigated the associations between the *Fixed and Adaptive Mode* measures of all CoGames. Using partial Spearman correlation, we found strong—very strong correlation estimates (range |*ρ*| = 0.57–0.89) between the two modes across all games, except for the game *Treasure Hunt*, where the correlation coefficient showed a moderate correlation (|*ρ*| = 0.48). All correlation estimates between modes can be found in Table 7.

**Table 7:** Partial spearman correlation coefficient estimates and 95% confidence intervals between primary measures of the fixed and adaptive mode.

Domain	Game in the *Fixed Mode* (measure)	Game in the *Adaptive Mode*^a^ (measure)	Spearman correlation coefficient [95% CI]	*n* PwMS
Working memory	Match maker (completion time)	Match maker (successful responses)	−0.75 [−0.84, −0.63]	100
Information processing speed	Think fast (completion time)	Think fast (successful responses)	−0.57 [−0.7, −0.41]	100
Information processing speed	Numbers (completion time)	Numbers (successful responses)	−0.85 [−0.9, −0.77]	100
Short-term memory	Treasure hunt (mean % of correct connections over 6 adaptive rounds)	Treasure hunt (mean % of correct connections over 6 adaptive rounds)	0.48 [0.3, 0.62]	100
Mental flexibility (set-shifting)	Mixer (completion time)	Mixer (successful responses)	−0.72 [−0.82, −0.58]	100
Psychomotor speed	Catch a Cloud (successful responses in 20 seconds)	Catch a Cloud (successful responses in 30 seconds)	0.89 [0.83, 0.93]	100

a Average of the first 2 runs.

### 3.4 Adherence

During the first 6 weeks, the average adherence across all CoGames was 89.3% (range: 86.6%–92.0%). As for the other tests included in the dreaMS app, the average adherence across all movement and balance tests was 79.7% (range: 71.2%–85.0%) and 83.2% (range: 82.0%–84.3%) for the vision tests. The adherence rates of all games and tests are shown in Table 8.

**Table 8:** Adherence rates of the first 6 weeks of all gamified (CoGames) and other tests included in the dreaMS app.

Game/test		Domain	Mean adherence	*n* pwMS
CoGames (high gamification)	Match maker	Working memory	89.0%	100
	Think fast	Information processing speed	89.2%	100
	Numbers	Information processing speed	89.2%	100
	Treasure hunt	Short-term memory	86.6%	100
	Mixer	Mental flexibility	89.6%	100
	Catch a cloud	Psychomotor speed	92.0%	100
Other dreaMS tests	Short promenade	Movement (30-seconds walk)	76.3%	100
	Spanish stairs	Movement (climbing stairs)	71.2%	100
	Up and go	Movement (stand up and walk)	76.8%	100
	Musical Chairs	Movement (stand up and sit down)	81.6%	100
	Walk a line	Balance (heel to toe balance walk)	85.0%	100
	Butler	Balance (stand still with stretched out arms)	83.3%	100
	Penguin	Balance (stand still)	79.7%	100
	Confetti	Dexterity (finger to nose test)	84.0%	100
	Eagle eye	Vision (near acuity)	84.3%	100
	Fog	Vision (low contrast)	82.0%	100

Green ≥ 90%; turquoise ≥ 85%; blue ≥ 80%; yellow < 80%.

## 4 Discussion

There is a need for more convenient and practical monitoring of cognitive function, offering a comprehensive assessment of disease progression, in chronic diseases such as multiple sclerosis. Gamified domain-specific cognitive tests performed on smartphones show great potential for accurate and enjoyable assessment of cognitive functions with high temporal resolution.

In this first data look we focused on the test-retest reliability of the CoGames and their correlations to domain-corresponding neuropsychological paper-pencil tests in pwMS. We also compared two modes of the CoGames, a fixed mode and an adaptive mode and assessed adherence over 6 weeks. This was particularly important because the dreaMS feasibility study (23) included similar cognitive games, but not the in-house developed CoGames. As this data look is part of dreaMS Validation study 1, no confirmatory statistical tests were applied to preserve the study's power.

Nevertheless, the point estimates for test-retest correlations of all but one (*Treasure Hunt)* of the games were good-excellent (*r* = 0.79–0.91). Overall, the reference tests partial Spearman correlation estimates ranged from |*ρ*| = 0.28 to 0.66 across all games in the *Fixed Mode* and |*ρ*| = 0.23 to 0.60 in the *Adaptive Mode*. The two modes showed strong to very strong correlation estimates with one another, except for the game *Treasure Hunt*, where the correlation estimate was moderate. Lastly, on average, adherence rates for the CoGames were around 10% higher compared to the movement & balance tests and around 6% higher compared to the vision tests included in the dreaMS app.

### 4.1 Reliability

Consistent measurement outcome is a key requirement of any assessment or monitoring tool. All CoGames levels of the *Adaptive Mode* were tested for reliability in a previous study with healthy volunteers (36). There, test-retest correlations between two runs of the same level across all games, showed strong and statistically significant correlations. Those results are congruent with our current findings where the primary measure of all but 1 game (*Treasure Hunt)* indicated good to excellent reliability. The weak test-retest correlation of the game *Treasure Hunt* was caused by a combination of the game's primary measure (mean percentage of correct connections) and the low difficulty of the level we used for the analysis (Level 1: Beginner). In measures using percentage of correct responses, low difficulty can lead to ceiling effects, i.e., reaching 100%. This in turn prevents the ranking necessary for Spearman's correlation calculation, resulting in lower coefficients, even though the true correlation is high. This was also observed in our previous study with healthy volunteers, where this effect was shown only for the 2 easiest levels and disappeared in higher levels, where there was no ceiling effect (36). Since all other games use a combination of accuracy and speed as their measure, this ceiling-effect-caused issue does not apply to them. We exploratorily investigated alternative measures for *Treasure Hunt* that are not only accuracy based: “memorization time” and “response time” (Table 4). Both measures showed correlations comparable to the other games: 0.71 and 0.75, respectively. These results further support our previously discussed assumption.

Comparable literature on reliability testing of smartphone-based games as assessment tools shows similar results: Nicosia et al. (2023) investigated the reliability of a set of smartphone games assessing multiple cognitive domains in a cohort consisting of mildly cognitively impaired and healthy elderly persons (39). Their test-retest analyses using follow-ups after 6 and 12 months showed ICC above 0.8. Similarly, McLaren B. et al. (2020) conducted reliability analyses of smartphone-based cognitive games in a sample of persons with premanifest Huntington's disease (HD), manifested HD, and healthy controls (40). They found moderate to strong ICCs (0.52–0.96). However, it is important to note that the high variability of study designs (e.g., frequency of completion, number of runs), statistical analyses, instruments used (i.e., platforms, models, software) and target groups (pwMS, healthy volunteers, mild cognitive impairment, HD, etc.) across studies, makes direct comparisons difficult. In our study we assessed the first 2 runs of the *Adaptive Mode* comprising the easiest difficulty level “Beginner” only. The mid- and longer-term reliability of other difficulty levels need to be performed using data of later stages of the study.

### 4.2 Associations between CoGames measures and reference tests

The goal of this analysis is not to show perfect correlations between established NPTs and our in-house developed monitoring tool, since we do not aim to copy, but rather improve cognitive monitoring. Nevertheless, a certain degree of concurrent validity (correlation to established reference tests) must be demonstrated in order to prove that the targeted cognitive domains can be assessed using this novel approach (Table 1).

In the *Fixed Mode* all but the game *Treasure Hunt* [*ρ* = 0.28 (0.07, 0.47)] showed moderate-strong correlation estimates with at least one of their reference tests. Considering only the stronger correlation, if 2 reference tests were used, the estimates ranged from |*ρ*| = 0.4 [0.21, 0.56] to 0.66 [0.53, 0.76]. The relatively low correlation estimate of *Treasure Hunt* was unexpected, particularly considering the higher estimate observed in the adaptive mode, which is more prone to yield ceiling effects. This warrants further investigation in the main analysis using a larger sample and additional timepoints. Similarly, correlation magnitudes between the CoGames in the *Adaptive Mode* and the reference tests were moderate-strong except for one game: *Match Maker* [*ρ* = 0.23 (0.03, 0.42)]. They ranged from |*ρ*| = 0.41 [0.21, 0.57] to 0.6 [0.45, 0.72]. The low correlation observed between *Match Maker* and the backward digit span may be partly explained by differences in the underlying performance measures. Although both tasks assess working memory, *Match Maker* focusses on speed, whereas the backward digit span is based on accuracy. At low difficulty levels, the game task may be further shifted toward a speed-dominated measure, as reduced cognitive demand minimizes working memory load. This interpretation is supported by the higher correlation observed in the *Fixed Mode*, which incorporates multiple difficulty levels and therefore places greater demands on working memory.

Interestingly, when cross correlating all games with all NPTs, some CoGames measures correlated similarly with NPTs of other cognitive domains, as with their reference test. Especially the TMT and SDMT showed correlation estimates with all games and both modes comparable to the corresponding reference test. Since the measures of all games, except for *Treasure Hunts,* are *time needed to complete the task* and the TMT A and SDMT are processing speed tests, correlation coefficients might be stronger because of this shared denominator. Furthermore, IPS has been shown to often be the first and most commonly impaired cognitive domain in pwMS (41, 42), frequently occurring before or concurrently with other cognitive deficits (43). This was also shown in our previous study, where different cognitive smartphone games were correlated with established NPTs (23). Thus, it is plausible that if other domains show deficits, IPS does so too. Of course, our results are only point estimates since this preliminary data analysis does not allow inferential statistics. Nevertheless, these estimates suggest that, although we see a certain degree of domain specificity, there is a strong influence of IPS on the assessment of all domains. To minimize this general effect, we assessed other game-derived measures such as error-rates. However, they were not suitable for the correlation analysis due to the very low error rate (ceiling effect). For the final dreaMS VS1 analysis we propose to correct the game measures of *Match Maker* and *Mixer* for IPS or reaction time, by subtracting from- or dividing by the measures of *Numbers, Think Fast*, or *Catch a Cloud*, similarly to the method sometimes applied in the Trail Making Test.

Notably, NPTs assessing the same cognitive domain (i.e., SDMT & TMT A) did not show stronger correlations with each other than with the CoGames measures (Supplementary Figures S2, 3). This further supports our assumption that our point estimates between the CoGames and the reference tests are strong enough to show a clear association. Especially considering that at this stage our goal is not to replace established NPTs, but rather to offer an additional level of sensitivity to monitoring cognition in MS, by increasing the temporal resolution of cognitive assessment in a more convenient, cost effective and enjoyable way.

### 4.3 Differences between the fixed and adaptive mode

Overall, the correlation estimates between the domain-specific reference tests and the CoGames showed similar results in both modes. Interestingly, *Match Maker* correlated weaker with its reference test in the *Adaptive Mode* than in the *Fixed Mode*. One reason for this might be the difficulty of the modes: The *Adaptive Mode* data consists of the average score of the two first runs, which are both in the easiest difficulty level. The *Fixed Mode* on the other hand, is designed to go through multiple difficulties from easy to hard. We assume that for simpler tasks, like the easier levels of *Match Maker*, the assessment shifts away from measuring working memory and more towards IPS. This might explain the lower correlation estimate with the reference test BDS, since its task is rather difficult and not time-based.

Across all games correlation estimates between the primary measures of the *Fixed Mode* and the *Adaptive Mode* of the same game showed strong to excellent correlations (range *ρ* = 0.57–0.89). Only in *Treasure Hunt* there was a moderate correlation (*ρ* = 0.48) (see Table 7), likely caused by the strongly skewed distribution of performance in the *Adaptive Mode*, which in turn is a result of the ceiling effect (task too easy). These findings suggest that the *Fixed Mode* and the *Adaptive Mode* are comparable, at least regarding the easiest difficulty level. As a follow-up it would be valuable to investigate the association between the *Fixed Mode* and the *Adaptive Mode* after patients reached the level corresponding to their performance. Then, correlation analyses might be more appropriate and representative using the patients’ performance-adapted level, as this would minimize the risk of a ceiling effect (i.e., skewed distribution) and prevent inaccurate assessment of maximum performance due to the patient still being in the learning phase. Additionally, since the *Adaptive Mode* uses randomized items, the average of multiple runs after the learning phase might give some stability to the game scores. Of course, the problem of comparing scores of levels with different difficulties remains. To address this issue, we might consider applying the formula proposed in the CoGames study which standardizes game scores across difficulty levels (36). This formula for a standardized score includes the raw score (primary measure of the game), predicted score (score based on the linear mixed model of the study population, depending on the level, age, operating system, and years of education), and the total variance (variance of the prediction + residual variance):

$$\mathrm{Standardized}\;\mathrm{score}=\frac{(\mathrm{raw}\;\mathrm{score}\;-\;\mathrm{predicted}\;\mathrm{score})}{\sqrt{\mathrm{total}\;\mathrm{variance}}}$$

The standardized score enables the comparison of scores of different difficulty levels, based on real-life data.

Comparable studies, investigating the validity of digital and gamified cognitive assessment tools in pwMS have shown similar results to ours: a study by Hsu et al. (2021) showed correlations between their tablet-based digital test battery: Adaptive Cognitive Evaluation “ACE” and the SDMT (*r* = –0.57, *p* < .001) (20). In another study by Hsu et al. (2021), the iPad-based action game “EVO-monitor” correlated significantly with the SDMT (*r* = 0.52, *p* < .001) and was able to differentiate pwMS from HC (21). The results of this analysis also support one of our prior proof-of-concept studies including 31 pwMS and 31 HC, where we also found moderate-strong correlations between cognitive training games and domain-respective NPTs (|*ρ*| = 0.34–0.77) (23). There, only 1 out of 10 games did not show statistically significant correlation to the domain corresponding reference tests which covered IPS, working memory, visuo-spatial construction- and short-term memory, inhibition, mental flexibility, and language. As discussed above, we observed the same influence of speed as a test-and game measure and general role of IPS regarding correlations between NPTs and cognitive smartphone games in pwMS. While the results found in the proof-of-concept study were promising, there were limitations that we were able to address in the current analysis. First, the study population is substantially larger in this analysis: 31 pwMS & 31 HC in the proof-of-concept study and 100 pwMS in this analysis, enhancing statistical power and generalizability. Second, in the previous study we did not account for change in difficulty level, potentially enabling biases when comparing measures across levels. Furthermore, we did not have access to the raw performance data and had to rely on system-generated scores influenced by gamification elements, such as multipliers for consecutive correct responses. Consequently, for some games we used the measure “maximum difficulty level reached” as an alternative measure of performance. While informative on performance, the granularity was reduced. In this analysis we focused on fixed difficulty levels and utilized raw scores as primary measures improving methodological rigor.

### 4.4 Adherence

The mean adherence (tests completed/tests scheduled) across all CoGames was 89.3%. The game *Treasure Hunt* showed the lowest adherence with 86.6%. We suspect that this is a result of the gameplay and feedback-design of the game: contrary to the other games, *Treasure Hunt* is not time-limited and might therefore last longer than 1 minute, which can be more exhausting. Furthermore, the feedback is binary (all or nothing) for each of the 6 rounds: the user is presented with either a green tick and a difficulty-dependent amount of points if the round was 100% correct or a red X and 0 points if the round was not 100% correct. Therefore, even if the recall was fairly good but not 100% correct, the user would receive 0 points. This feedback system increases the risk of frustration. Supporting this hypothesis is data from our previous work (33) where participants were asked to rate all games regarding frustration. There, *Treasure Hunt* was rated the most frustrating game, with positive correlation between the frustration rating and the difficulty level.

The mean adherence differences between the other dreaMS tests and the CoGames were very clear: on average, across all tests and games, participants were around 10% more adherent playing the games compared to the movement & balance tests, and around 6% more adherent compared to the vision tests. In combination with findings of our previous studies, these results support our hypothesis that more enjoyable and motivating active tests are more consistently completed by pwMS. Of course, this analysis only looks at the first 6 weeks. Long-term development and change in adherence for both the gamified and other tests might differ and must be investigated after a longer period.

### 4.5 Limitations

The reported analysis has some limitations: Firstly, this is a data look analysis with limited sample size and follow-up. It includes only the first 100 patients, which completed the first 6 weeks (intensive phase) of the dreaMS VS1. This relatively small sample is more vulnerable to missing values, leading to a reduced n for some of the analyses. Furthermore, we acknowledge a potential selection bias towards more physically and cognitively fit individuals, who experience less fatigue and are more familiar with digital tools. Then, 6 weeks were not enough to reach the adequate difficulty level resulting in patients being in different levels for the analysis. To avoid comparing across multiple difficulty levels we used the average score of only the first 2 runs for the correlations analyses, where all participants were in the same level (Beginner). Therefore, all patients played the easiest level, which for many led to ceiling-effects in the game *Treasure Hunt*. Of course, to investigate whether or not the *Adaptive Mode* and its dynamic difficulty adjustment system adapt properly to the patients performance and offer better detection of meaningful change, further analyses over longer periods must be conducted. The selection of NPT as references was also limited, since the neuropsychological testing was part of the Swiss MS Cohort study which used a fixed test battery. While we were able to find NPTs assessing the same cognitive domain, the measure used (e.g., speed-dependent) has an impact on correlation, as was shown in this analysis. In order to minimize the influence of IPS on the assessment of other domains, speed-independent games would have to be developed. However, we purposely designed the games with time-limits to reduce inconvenience and fatigue for the patients who are asked to regularly play them. Further, the majority of patients show no signs of cognitive impairment which reduces the chance of finding strong correlations. A group differentiation analysis was not possible at this stage, because our current data set did not yet contain a sufficient number of healthy volunteer data. However, we plan to investigate differences between healthy controls and pwMS in the main dreaMS VS1 analysis. Lastly, because this is a data look not justifying inferential statistics, the point estimates provided are only indicative and true CI or even p-values cannot be estimated. Our findings regarding reliability, correlation to established NPTs, and adherence must be confirmed in the main dreaMS VS1 analysis. Additional analyses on potential practice effects, the dynamic difficulty adjustment system, and group differentiation (healthy vs. pwMS) are also needed.

### 4.6 Conclusions

In this subsample-analysis of the dreaMS Validation study 1 we investigated the test-retest reliability, correlation to established reference tests, and adherence rates of cognitive smartphone games (CoGames) as a monitoring tool for cognition in pwMS. Our data increases confidence that all our in-house developed CoGames are reliable, show moderate to strong correlation estimates to their domain-corresponding predefined reference test in at least one of the two modes, and that patients are more adherent playing the games than completing the other dreaMS tests. Together with the results of two previous studies supporting these findings (23, 36) we gained confidence that CoGames has the potential to become a convenient and enjoyable method to monitor cognitive function in pwMS and if further validated, in other chronic diseases affecting cognitive function. To our knowledge, CoGames is the only monitoring tool that combines domain-specific, rigorous, and high-frequency monitoring of cognitive functions, gamification elements to enhance motivation, engagement, and ultimately adherence, and an adaptive difficulty system, which challenges the users but avoids frustration, which is especially relevant in diseases with progressive cognitive decline.

## Data Availability

The datasets presented in this article are not readily available because the data is preliminary from an ongoing study. Requests to access the datasets should be directed to silvan.pless@usb.ch.
