# Supplementary figures and images for: Monitoring cognition in multiple sclerosis via adaptive smartphone games—first insights from a validation study
Source: Front Digit Health. 2026 Feb 10;8:1627226. doi: 10.3389/fdgth.2026.1627226 (PMC12930360; doi:10.3389/fdgth.2026.1627226)

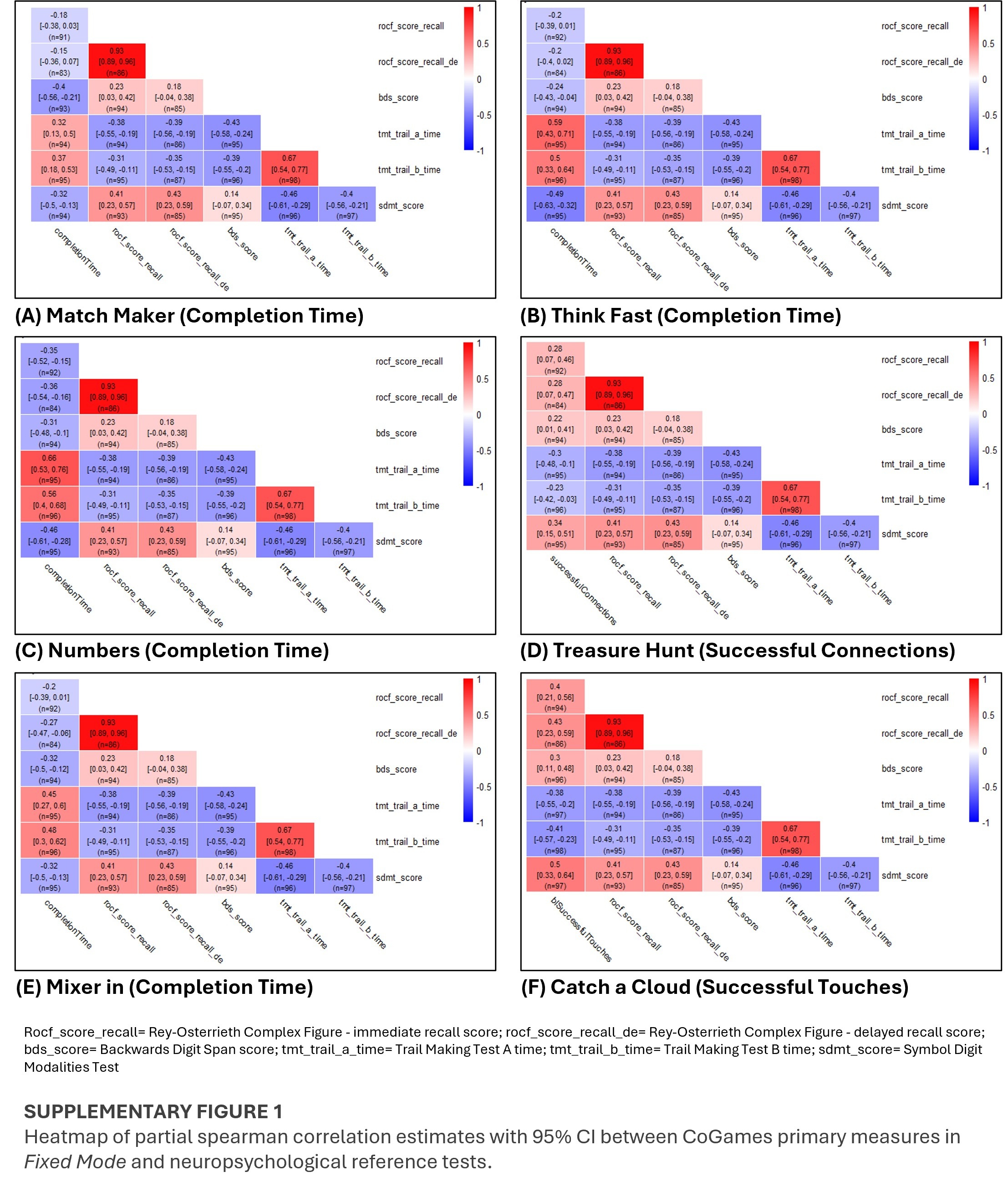

Supplement: Supplementary file 1 [file Image1.jpg]

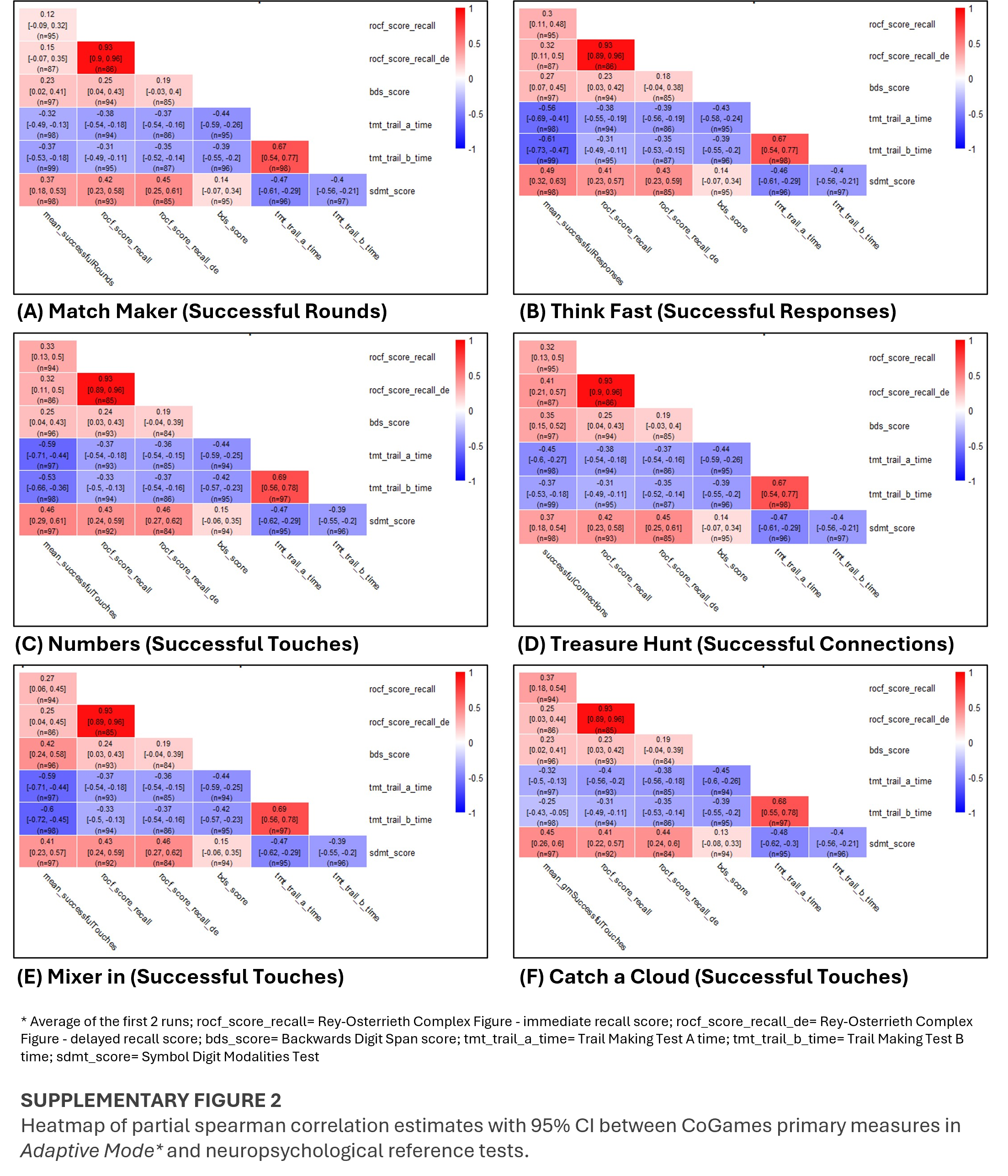

Supplement: Supplementary file 2 [file Image2.jpg]
